# Infiltrating myeloid cell-derived properdin markedly promotes microglia-mediated neuroinflammation after ischemic stroke

**DOI:** 10.1186/s12974-023-02946-z

**Published:** 2023-11-11

**Authors:** Pin-yi Liu, Hui-qin Li, Meng-qi Dong, Xin-ya Gu, Si-yi Xu, Sheng-nan Xia, Xin-yu Bao, Yun Xu, Xiang Cao

**Affiliations:** 1 Department of Neurology, Nanjing Drum Tower Hospital, Affiliated Hospital of Medical School, Nanjing University, 321 Zhongshan Road, Nanjing, Jiangsu 210008 People’s Republic of China; 2 Department of Neurology, Nanjing Drum Tower Hospital, State Key Laboratory of Pharmaceutical Biotechnology and Institute of Translational Medicine for Brain Critical Diseases, Nanjing University, Nanjing, Jiangsu 210008 People’s Republic of China; 3 Jiangsu Key Laboratory for Molecular Medicine, Medical School of Nanjing University, Nanjing, Jiangsu 210008 People’s Republic of China; 4 Jiangsu Provincial Key Discipline of Neurology, Nanjing, Jiangsu 210008 People’s Republic of China; 5 Nanjing Neurology Medical Center, Nanjing, Jiangsu 210008 People’s Republic of China

**Keywords:** Ischemic stroke, Microglia, Properdin, Macrophages, Neutrophils, Mincle

## Abstract

**Background:**

Emerging evidence has shown that myeloid cells that infiltrate into the peri-infarct region may influence the progression of ischemic stroke by interacting with microglia. Properdin, which is typically secreted by immune cells such as neutrophils, monocytes, and T cells, has been found to possess damage-associated molecular patterns (DAMPs) properties and can perform functions unrelated to the complement pathway. However, the role of properdin in modulating microglia-mediated post-stroke neuroinflammation remains unclear.

**Methods:**

Global and conditional (myeloid-specific) properdin-knockout mice were subjected to transient middle cerebral artery occlusion (tMCAO). Histopathological and behavioral tests were performed to assess ischemic brain injury in mice. Single-cell RNA sequencing and immunofluorescence staining were applied to explore the source and the expression level of properdin. The transcriptomic profile of properdin-activated primary microglia was depicted by transcriptome sequencing. Lentivirus was used for macrophage-inducible C-type lectin (Mincle) silencing in microglia. Conditioned medium from primary microglia was administered to primary cortex neurons to determine the neurotoxicity of microglia. A series of cellular and molecular biological techniques were used to evaluate the proinflammatory response, neuronal death, protein–protein interactions, and related signaling pathways, etc.

**Results:**

The level of properdin was significantly increased, and brain-infiltrating neutrophils and macrophages were the main sources of properdin in the ischemic brain. Global and conditional myeloid knockout of properdin attenuated microglial overactivation and inflammatory responses at the acute stage of tMCAO in mice. Accordingly, treatment with recombinant properdin enhanced the production of proinflammatory cytokines and augmented microglia-potentiated neuronal death in primary culture. Mechanistically, recombinant properdin served as a novel ligand that activated Mincle receptors on microglia and downstream pathways to drive primary microglia-induced inflammatory responses. Intriguingly, properdin can directly bind to the microglial Mincle receptor to exert the above effects, while Mincle knockdown limits properdin-mediated microglial inflammation.

**Conclusion:**

Properdin is a new medium by which infiltrating peripheral myeloid cells communicate with microglia, further activate microglia, and exacerbate brain injury in the ischemic brain, suggesting that targeted disruption of the interaction between properdin and Mincle on microglia or inhibition of their downstream signaling may improve the prognosis of ischemic stroke.

**Supplementary Information:**

The online version contains supplementary material available at 10.1186/s12974-023-02946-z.

## Background

Ischemic stroke is a major cause of high mortality and disability worldwide [1, 2]. Numerous studies indicate that persistent inflammatory responses mediated by immune cells, including brain-resident microglia and peripheral infiltrating cells, can cause secondary brain injury [3–5]. Among peripheral cells, neutrophils and macrophages are the first immune cells that infiltrate brain tissue and modulate inflammatory responses during ischemic stroke [6, 7]. Previous research by our laboratory and others has partially shed light on the crosstalk between microglia and infiltrating myeloid cells. Microglia can regulate neutrophil infiltration, and conversely, neutrophils might modulate the polarization of microglia [8–10]. However, little attention has been paid to the interaction between microglia and macrophages, primarily due to the difficulty in distinguishing them based on their similar embryonic origin and cellular markers.

Properdin, a plasma protein encoded by the *Cfp* gene, positively regulates the complement pathway and is derived from various inflammatory cells, including monocytes, dendritic cells, neutrophils, and T cells [11–13]. In addition to its classical function in stabilizing C3 and C5 convertases, properdin has multifaceted roles in various diseases that are independent of its role in the complement system [14]. Properdin can act as an inducible damage-associated molecular pattern (DAMP), activating NK cells and lymphoid cells [15, 16] and labeling apoptotic or necrotic cells to assist phagocytes in finding and clearing damaged cells [17, 18]. During ischemic insult, all types of dying cells can release DAMPs, which bind to pattern recognized receptors (PRRs) on microglia, initiating a cascade of inflammatory signaling pathways and leading to the production of inflammatory cytokines, including IL-1β, TNF-α, and IL-6. This exacerbates additional neuronal death and ischemic brain damage. The concentration of properdin in healthy human sera is nearly 20 µg/ml, and it is present at a low level in cerebrospinal fluid [19, 20]. Given the blood–brain barrier (BBB) dysfunction that occurs during ischemic stroke, properdin might cross the BBB and enter the brain. Although properdin has been shown to be involved in proinflammatory responses in diseases such as asthma and tuberculosis [21, 22], it is unknown whether properdin influences brain immunity, inflammation and prognosis in ischemic stroke.

In this work, we explored the role and mechanism of properdin in ischemic stroke. By using both global and conditional (myeloid-specific) properdin-knockout mice, we found that macrophage- and neutrophil-derived properdin was detrimental to neurological outcomes owing to its ability to activate microglia. Through transcriptomics and molecular experiments, we discovered that the DAMP properdin could bind to macrophage-inducible C-type lectin (Mincle) on microglia, a member of the C-type lectin receptor family, and activate multiple downstream inflammatory pathways. Inhibition of Mincle effectively reduced the proinflammatory responses induced by properdin in microglia. Our findings highlight a novel function of properdin in the microglia-dependent inflammatory response in ischemic stroke, where it acts as a DAMP that exacerbates neuroinflammation. This study provides a potential therapeutic target for modulating the crosstalk between peripheral and brain immunity in ischemic stroke.

## Materials and methods

### Mouse strains and genotyping

Male C57BL/6J (B6) mice and LysM-cre mice were purchased from the Model Animal Research Center of Nanjing University (Nanjing, China). The *Cfp* knock out (*Cfp*^−/−^) and floxed *Cfp* (*Cfp*^fl/fl^) mice on C57BL/6J genetic background were generated by Cyagen Biosciences (Guangzhou, China). *Cfp*^fl/fl^ mice were crossed with LysM-cre mice to generate myeloid-specific *Cfp* knock out mice. PCR-based genotyping was performed on mouse DNA. The following primers were used for genotyping of *Cfp*^−/−^, *Cfp*^fl/fl^ and LysM-cre mice: *Cfp*^−/−^-upper 5ʹ-TACTGCATACAGACCTAAAGCTGC-3ʹ, *Cfp*^−/−^-lower1 5ʹ-TGTTGGACTTAGTCTGCTTTCAGT-3ʹ, *Cfp*^−/−^-lower2 5ʹ-GAGACAAATTCTCACTGAAGGGCA-3ʹ. *Cfp*^fl/fl^-upper 5ʹ-AATGCTTTAAAAGTCAGGGCTTCC-3ʹ, *Cfp*^fl/fl^-lower 5ʹ-TTTGGCATTGTCCTCAGAATTTCC-3ʹ. LysM-cre-upper 5ʹ-CCCAGAAATGCCAGATTACG-3ʹ, LysM-cre-lower1 5ʹ-CTTGGGCTGCCAGAATTTCTC-3ʹ, LysM-cre-lower2 5ʹ-TTACAGTCGGCCAGGCTGAC-3ʹ. All experiments on mice were performed according to the Guide for the Animal Care and Use Committee of Nanjing University.

### tMCAO and laser speckle contrast imaging (LSCI)

8-week-old male mice were subjected to tMCAO with the intraluminal filament technique as previously described [23]. In brief, after being deeply anesthetized with tribromoethanol, the right common carotid artery and external carotid artery were isolated via the mid-neck incision. Next, a 6-0 silicon-coated monofilament suture was inserted into the internal carotid artery via the external carotid artery and left in situ for 60 min. LSCI was conducted through the intact skull to visualize the cerebral blood flow during occlusion and reperfusion and regions of interest (ROIs) were selected for the following quantitative analysis.

### Single-cell RNA sequencing (scRNA-seq)

C57BL/6J mice from the tMCAO and sham groups were subjected to scRNA-seq. Sham mice and tMCAO model mice were euthanized at 3 h, 12 h and 3 days after tMCAO. Subsequent sample processing and scRNA-seq were conducted by OE Biotech Co., Ltd. (Shanghai, China). Briefly, the single-cell cDNA library was constructed based on the single-cell samples followed by quality assessment. The filtered gene-barcode matrix was normalized and processed for dimensionality reduction visualized by t-distributed stochastic neighbor embedding (t-SNE) using the R package (Version 3.0). The expression level of *Cfp* was visualized by Loupe Browser 5 (Version 5.1.0, 10× Genomics).

### Behavioral tests

A battery of behavioral tests including the modified neurological severity score (mNSS), grip strength and rotarod test was used to assess the functional deficits of tMCAO mice. The behavioral tests were performed by an investigator blinded to experimental groups. mNSS was graded on a scale of 0 to 18 and involves assessment of motor, sensory, reflex, and balance. A higher score indicates more severe deficit. Forelimb grip strength was measured using a grip strength meter (GS3, Bioseb, France). Each mouse was held by the tail and the forepaws were allowed to grasp the T-bar of the strength meter five times, and the maximum grip was recorded. In the rotarod test, mice were forced to run on a rotarod cylinder (RWD Life Science, Shenzhen, China) at a linear acceleration from 5 to 40 rpm/min within 5 min. The duration for each mouse on the device was recorded as the latency to fall.

### Infarct volume evaluation

The infarct volume was measured using 2% 2,3,5-triphenyltetrazolium chloride (TTC, Sigma-Aldrich) staining. After being deeply anesthetized, the mice were euthanized to isolate the brains, which were then coronally sectioned into 5 slices with 2 mm thickness. Then the brain sections were incubated with 2% TTC in PBS at 37 °C for 10 min. The infarct areas appeared pale grey while the normal regions were stained red. The infarct volume was analyzed by ImageJ software (USA, RRID:SCR_003070) and calculated as follows: (contralateral hemisphere area − noninfarct ipsilateral hemisphere area)/(2 × contralateral hemisphere area) × 100%.

### Primary microglia and primary cortical neuron culture

Primary microglia were isolated from brains of neonatal C57BL/6J (B6) mice (P0–P1) as previously described [24]. Briefly, after stripping the meninges, the cerebral cortex was dissociated using mechanical shearing and TrypLE. Then, the cells obtained from one brain were plated on a T75 flask and cultivated in DMEM supplemented with 10% fetal calf serum and 1% penicillin/streptomycin. After 10 days, the culture flasks were gently tapped and the floating microglia were collected for subsequent reseeding in the indicated well plates for subsequent experiments.

Primary cortical neurons were prepared from E15-17 embryos according to a previous protocol [25]. Cerebral cortices were isolated in ice-cold DMEM and then treated with TrypLE. After repeated pipetting up and down, the resulting cells were plated on poly-d-lysine-coated plates. Primary neurons were cultured in neurobasal medium containing B27, GlutaMAX and penicillin/streptomycin. For cell viability assay, neurons were seeded in 96-well plates and incubated with different concentrations of eukaryotic rmProperdin (Cusabio, Wuhan, China)-treated microglial conditioned media for 12 h. Subsequently, 10 μl of CCK8 solution (MedChemExpress, NJ, USA) was added to each well at 37 °C. Finally, the absorbance at 450 nm was measured using a microplate reader (Tecan Trading AG, Switzerland).

### Lentiviral knockdown of *Clec4e*

*Clec4e* knockdown in primary microglia was achieved by using lentivirus shRNA targeting the *Clec4e* gene purchased from GeneChem (Shanghai, China). Non-silencing lentivirus shRNA was used as control. Primary microglia were seeded into 6/12-well plates and infected with lentivirus (MOI = 40) in the presence of 10 μg/ml polybrene. The infection medium was replaced after 8 h, and microglia continued to be cultured for 3 days for the following experiment. qPCR and Western blotting were used to determine the knockdown efficiency.

### Fluorescence-activated cell sorting (FACS) and flow cytometry analysis

Mice subjected to tMCAO were transcardially perfused with cold PBS, and ischemic hemispheres were harvested for subsequent microglia extraction. Then, the brain tissues were minced and homogenized in cold DMEM, and the resulting cloudy suspensions were filtered through a 70-µm cell strainer. To remove the myelin debris fraction, the single-cell suspensions were separated on a 30%–70% Percoll gradient, and the cells were isolated for subsequent FACS analysis.

The isolated cells were then stained with fluorophore-labeled anti-mouse CD45 (Rat, 1:1000, BioLegend, CA, USA, Cat# 103114, RRID: AB 312979) and CD11b (Rat, 1:500, Thermo Fisher Scientific, OH, USA, Cat# 53-0112-82, RRID: AB 469901) antibodies for 30 min at 4 °C, and the CD45^int^CD11b^+^ cells were sorted by BD FACSAria™ III (San Jose, CA, USA) and considered microglia. PCR array analysis was further performed on microglia.

To evaluate the levels of inflammatory factors in microglia in vivo and in vitro, the cells were further incubated with the following antibodies: PE-anti-TNF-α (Rat, 1:200, Thermo Fisher Scientific, OH, USA, Cat# 12-7321-81, RRID:AB_466198), PE-anti-IL-6 (Rat, 1:200, BioLegend, CA, USA, Cat# 504503, RRID:AB_315337) and APC-anti-IL-1β. (Rabbit, 1:500, Cell Signaling Technology, MA, USA, Cat# 31202, RRID:AB_2799001) All the samples were analyzed by a BD LSRFortessa flow cytometer (BD, NJ, USA), and the data were further analyzed using FlowJo software (Ashland, OR, USA, RRID:SCR_008520).

### PCR array analysis

The inflammatory cytokine and receptor PCR array assay was conducted according to the manufacturer’s instructions (Wcgene Biotech, Shanghai, China). Four ischemic hemispheres were pooled into one sample, and microglia were isolated by FACS. Total RNA was extracted from microglia and reverse-transcribed into cDNA for subsequent PCR array analysis. The data were analyzed using Wcgene Biotech software.

### RNAseq analysis of primary microglia

Three samples each of control primary microglia and 8 µg/ml properdin-treated primary microglia were subjected to RNAseq analysis. Total RNA was extracted using TRIzol reagent, and the RNA quality and quantity were ensured by a NanoDrop 2000 spectrophotometer (Thermo Scientific, USA) and Agilent 2100 Bioanalyzer (Agilent, Santa Clara, CA, USA). Transcriptome sequencing and analysis were conducted by OE Biotech Co., Ltd. (Shanghai, China). Total RNA-seq libraries were constructed using the TruSeq Stranded mRNA LTSample Prep Kit (Illumina, San Diego, CA, USA) and sequenced on the Illumina sequencing platform (HiSeqTM 2500 or Illumina HiSeq X Ten). Then, the obtained raw reads were processed using Trimmomatic to require clean reads for subsequent analysis. Differentially expressed genes (DEGs) were defined by a false discovery rate [FDR] < 0.05 and a |fold change|> 2. Hierarchical cluster analysis and volcano plots of the DEGs were conducted to identify gene expression patterns in different groups. GO enrichment analysis, KEGG pathway enrichment analysis and GSEA were carried out to further analyze the DEGs.

### Western blotting

The ischemic hemispheres after perfusion with PBS or primary microglia were harvested and lysed in RIPA buffer containing protease inhibitor cocktail. Equal amounts of protein (40 µg of protein from the ischemic hemisphere or 20 µg of protein from primary microglia) were electrophoretically separated on SDS-PAGE gels, and transferred to PVDF membranes (Millipore, USA). After being blocked in 5% skim milk, the membranes were incubated with specific primary antibodies overnight at 4 °C. The primary antibodies included anti-properdin (Mouse, 1:100, Santa Cruz Biotechnology, CA, USA, Cat# sc-393723, RRID:AB_3067070), anti-Mincle (Mouse, 1:200, Santa Cruz Biotechnology, CA, USA, Cat# sc-390806, RRID:AB_3067071), anti-p-Syk (Rabbit, 1:1000, Cell Signaling Technology, MA, USA, Cat# 2711, RRID:AB_2197215), anti-Syk (Rabbit, 1:1000, Cell Signaling Technology, MA, USA, Cat# 2712, RRID:AB_2197223), anti-p-p65 (Rabbit, 1:1000, Cell Signaling Technology, MA, USA, Cat# 3033, RRID:AB_331284), anti-p65 (Rabbit, 1:1000, Bioworld Technology, Nanjing, China, Cat# BS1257, RRID:AB_1663265), anti-C/EBPβ (Mouse, 1:200, Santa Cruz Biotechnology, CA, USA, Cat# sc-7962, RRID:AB_626772), anti-GAPDH (Rabbit, 1:5000, Bioworld Technology, Nanjing, China, Cat# AP0063, RRID:AB_2651132). The next day, the membranes were washed and incubated with the horseradish peroxidase (HRP)-conjugated secondary antibodies at room temperature (RT) for 2 h. The protein bands were visualized using a GEL-Pro system (Tanon Technologies, Shanghai, China) and analyzed by ImageJ (USA, RRID:SCR_003070) software.

### Enzyme-linked immunosorbent assay (ELISA)

Blood was collected from the sham-operation and tMCAO mice and clotted for 30 min followed by centrifugation to isolate serum. Properdin levels in serum after tMCAO were determined using properdin ELISA kit following the manufacturer’s guidelines (Signalway Antibody, MD, USA). The levels of properdin in the sham and ischemic hemispheres were also measured using ELISA after protein quantification. The standard curve was constructed using a four-parameter logistic (4-PL) curve-fit.

### Immunofluorescence staining

Twenty-micrometer-thick coronal brain sections were permeabilized with 0.25% Triton X-100 in PBS (PBST) at RT for 10 min and blocked with 2% donkey serum at RT for 1 h followed by overnight incubation with primary antibodies at 4 °C. The primary antibodies included anti-Ly6G (Rabbit, 1:200, Cell Signaling Technology, MA, USA, Cat# 87048, RRID:AB_2909808), anti-properdin (Mouse, 1:50, Santa Cruz Biotechnology, CA, USA, Cat# sc-365634, RRID:AB_10844832), anti-TMEM119 (Rabbit, 1:200, Abcam, Cambridge, UK Cat# ab209064, RRID:AB_2800343), and anti-Iba-1 (Rabbit, 1:500, Abcam, Cambridge, UK, Cat# ab5076, RRID:AB_2224402). Subsequently, the sections were washed for three times and then incubated with appropriate secondary antibodies at RT for 1 h. After washing with PBS, the sections were mounted with Antifade Mounting Medium with DAPI (Beyotime, China). To evaluate neuronal apoptosis in vivo, brain sections were subjected to a TUNEL assay before NeuN immunofluorescence labeling according to the manufacturer’s instructions (Vazyme, China). In vitro primary cortical neuronal apoptosis was assessed via Calcein-AM and PI dual labeling. Images were captured using confocal microscopy (Olympus FV3000, Japan) with a 40 × objective (20 × objective for TUNEL assay). The mean fluorescence intensity was analyzed by ImageJ. In the analysis of microglial morphology, a Z-stack with an interval of 1 µm was used. Three fields of interest were captured per mouse, and the analysis of all microglia within each field was conducted using MotiQ, a custom-made plugin for ImageJ [26]. The MotiQ thresholder was used to process the images for the following MotiQ 2D analyser to analyze the branch length, branch number and junctions of microglia. A MotiQ 3D analyser was used to generate 3D image visualizations of microglia.

### RNA isolation and quantitative PCR (qPCR)

Total RNA was prepared using TRIzol reagent as previously described and then equal amounts of RNA were reversed-transcribed into cDNA using HiScript III Q RT SuperMix for qPCR (Vazyme, Nanjing, China). qPCR was performed with AceQ qPCR SYBR® Green Master Mix (Vazyme) on Roche LightCycler 96 PCR instrument (Roche, Sweden). Relative gene expression levels were calculated using the 2^−∆∆Ct^ method and GAPDH was used as the endogenous control.

### Co-immunoprecipitation (Co-IP)

Total cell lysates from primary microglia or brain tissues were prepared with RIPA buffer containing protease inhibitors and pre-cleared with Protein A/G PLUS-agarose beads (Millipore, Billerica, MA, USA). Lysates containing 500 µg of total protein were incubated with 2 µg mouse anti-Mincle or control mouse IgG (Millipore, USA) overnight at 4 °C. Subsequently, Protein A/G PLUS-agarose beads were added to the mixture at 4 °C for 3 h to bind to the immune complexes. Finally, the protein complexes were eluted from the beads with SDS-PAGE loading buffer followed by immunoblotting with anti-properdin (Santa Cruz Biotechnology).

### Duolink proximity ligation assay (PLA)

Interactions of properdin with Mincle were detected and visualized using a Duolink proximity ligation assay kit (Sigma‒Aldrich). Properdin-treated and control primary microglia were fixed with 4% paraformaldehyde, permeabilized with 0.25% PBST and blocked with Duolink Blocking Solution. Then, the microglia were incubated with mouse anti-Mincle and rabbit anti-properdin antibodies at 4 °C overnight. After sequential incubation with the PLUS and MINUS PLA probes, ligase, and polymerase, the nuclei were stained with DAPI. The PLA signals were generated if the two probes were in close proximity (< 40 nm).

### Statistics analysis

The data in this study were normally distributed and are displayed as the mean ± standard error of the mean (SEM). All data were analyzed by SPSS 18.0 software. *P* values were determined as specified in the figure legends. A *P* value < 0.05 is considered statistically significant.

## Results

### Properdin accumulates in the ischemic brain and exacerbates brain injury

**Fig. 1:**
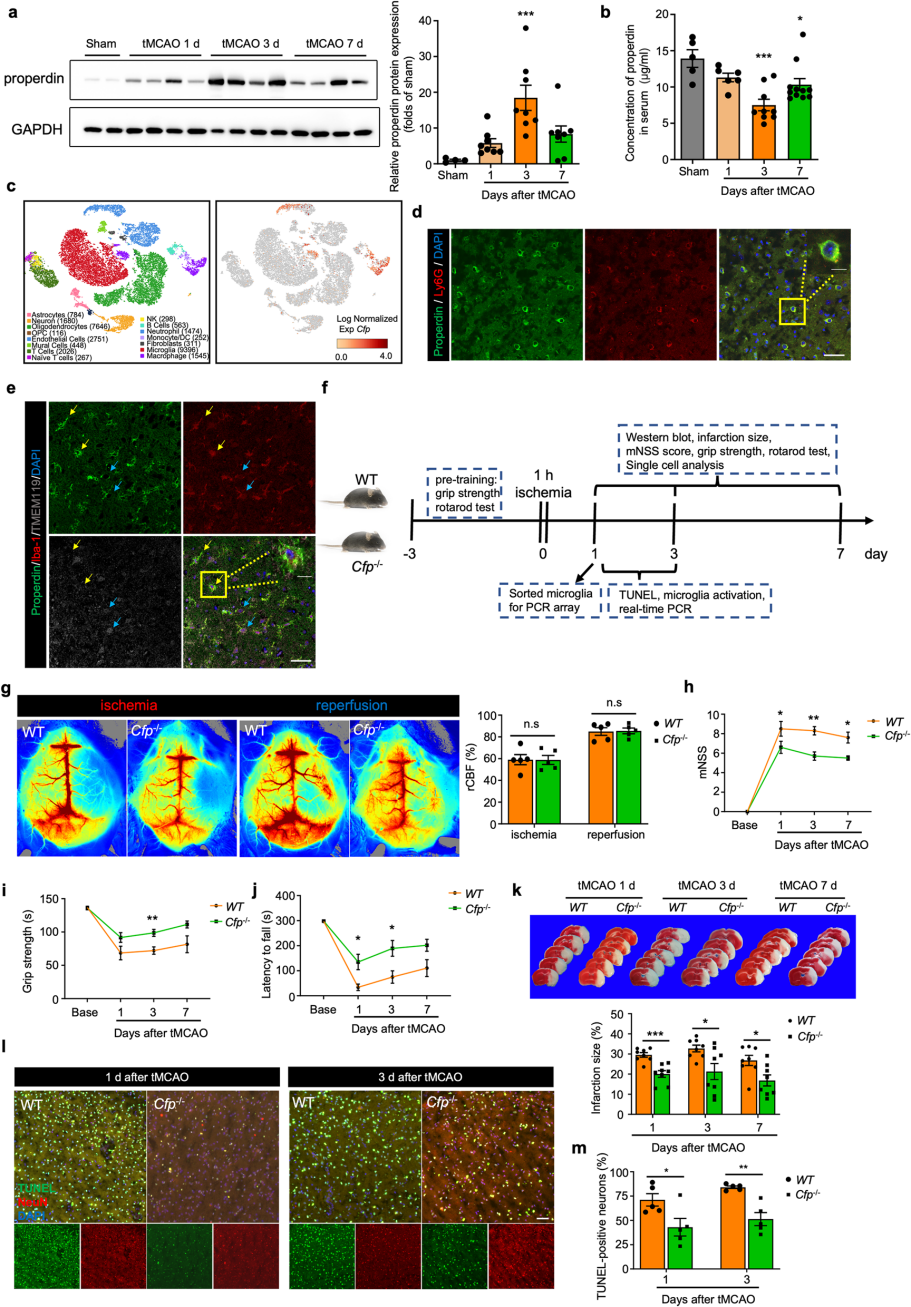
Properdin accumulates in the ischemic brain and exacerbates brain injury. **a** Protein expression of properdin relative to GAPDH in the ischemic hemisphere. *n* = 4 in sham group and 8 in the tMCAO group. ***P < 0.001 versus the sham group, one-way ANOVA with Bonferroni post hoc test. **b** Concentrations of properdin in the serum of tMCAO model mice. *n* = 5 in the sham group, 6 in the 1 d after tMCAO group, 9 in the 3 d after tMCAO group and 11 in the 7 d after tMCAO group. *P < 0.05 versus the sham group, ***P < 0.001 versus the sham group, one-way ANOVA with Bonferroni post hoc test. **c** The tSNE plot shows the clusters of brain cells (left, cell numbers were indicated in the parentheses) and visualizes the distribution of *Cfp* expression (right) after ischemic stroke. **d** Immunostaining for properdin and Ly6G in C57BL/6J (B6) mice 1 d after tMCAO. Scale bars: 10 µm and 50 µm. **e** Immunostaining for properdin, Iba-1 and TMEM119 in C57BL/6J (B6) mice 3 d after tMCAO. Yellow arrows: macrophages. Blue arrows: microglia. Scale bars: 10 µm and 50 µm. **f** Timeline of the experimental design. **g** Assessment of regional cerebral blood flow in WT and *Cfp*^−/−^ mice subjected to tMCAO. *n* = 5 mice per group. n.s, no significance (*P* > 0.05), two-way ANOVA with Sidak's multiple comparisons test. **h**–**j** Neurological deficits were evaluated by the mNSS (**h**), grip strength (**i**) and rotarod test (**j**) at 1 d, 3 d, and 7 d after tMCAO. *n* = 10 mice per group. *P < 0.05 versus the WT group, **P < 0.01 versus the WT group, two-way ANOVA with Sidak's multiple comparisons test. **k** The infarct area was assessed by TTC staining 1 d, 3 d, and 7 d after tMCAO. *n* = 8 mice per group. *P < 0.05 versus the WT group, ***P < 0.001 versus the WT group, unpaired Student’s *t* test. **l**, **m** Representative images and quantification of apoptotic neurons stained with TUNEL and NeuN 1 d and 3 d after tMCAO. Scale bar: 50 µm. *n* = 5 mice per group. *P < 0.05 versus the WT group, **P < 0.01 versus the WT group, unpaired Student’s *t* test

The role of properdin in ischemic stroke remains unclear. We first assessed the properdin levels in the brain and peripheral circulation 1 day (d), 3 d and 7 d after transient middle cerebral artery occlusion (tMCAO). In the ischemic hemisphere, properdin was significantly elevated from 1 to 3 d after tMCAO, while its level dropped at day 7 post-stroke but was still higher than its baseline (Fig. 1a, Additional file 1: Figure S1a). Intriguingly, the change of properdin level in the serum was exactly opposite to that in the brain. After tMCAO, circulating properdin levels decreased and reached their lowest levels on the 3rd day (Fig. 1b). These results suggested that properdin may migrate from the periphery to the brain by itself or via the cells that secrete it after ischemic stroke. In our recent single-cell RNA sequencing (scRNA-seq) analysis of the brains of tMCAO model mice (3 h, 12 h, and 3 d) and sham mice, 15 cell types were identified based on the expression of canonical markers. As shown in Fig. 1c and Additional file 1: Figure S1b, *Cfp* was mainly expressed in neutrophils and macrophages. Consistently, we further conducted immunofluorescence staining and confirmed that the *Cfp*-encoded protein properdin could be clearly detected in neutrophils and macrophages with a decreased gradient from the ischemic core to the distal areas. Moreover, properdin was almost not detected in microglia (Fig. 1d, e, Additional file 1: Figure S2).

The properdin levels in the brain and circulation were dramatically changed after ischemic stroke. However, the effect of this change on the outcome of ischemic stroke remains unclear. Thus, we used *Cfp*^−/−^ mice, which are characterized by the global knock out (KO) of the gene (*Cfp*) that encodes properdin (Additional file 1: Figure S3), and we assessed the neurological function and infarct volume in these mice after tMCAO (Fig. 1f). *Cfp*^−/−^ mice and their wild-type (WT) littermates were subjected to tMCAO and showed comparable regional cerebral blood flow (rCBF), indicating no differences in blood perfusion during the ischemia or reperfusion period (Fig. 1g). Neurological function deficits were evaluated at 1 d, 3 d, and 7 d after tMCAO. Compared to WT mice, *Cfp*^−/−^ mice had improved motor, sensory and balance functions, as revealed by decreased mNSS scores and increased time spent on a rod. Moreover, the forepaw grip strength assessment suggested that *Cfp*^−/−^ mice had greater muscle strength (Fig. 1h–j). In addition to the improvement in neurological function, *Cfp*^−/−^ mice exhibited decreased infarct volume compared with WT mice (Fig. 1k). We also performed a TUNEL assay to label apoptotic neurons and found that *Cfp*^−/−^ mice had fewer apoptotic neurons than WT mice (Fig. 1l, m). These results suggest that properdin is a detrimental factor that contributes to worsened ischemic brain injury.

### Properdin knockout ameliorates microglial activation and inflammation in tMCAO mice

**Fig. 2:**
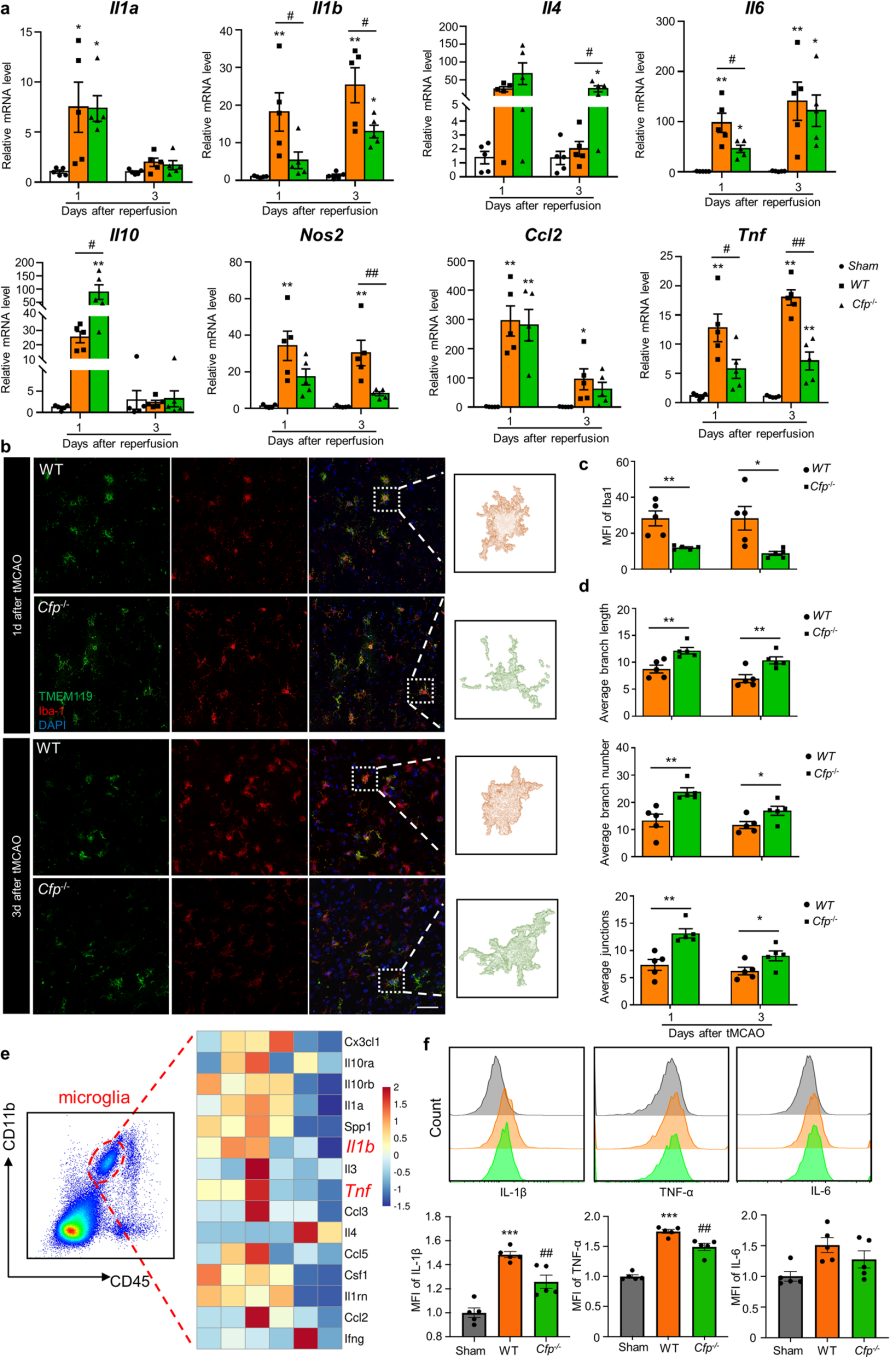
Properdin knockout ameliorates microglial activation and inflammation in tMCAO mice. **a** qPCR assessment of differentially expressed inflammatory genes, including *Il1a*, *Il1b*, *Il4*, *Il6*, *Il10*, *Nos2*, *Ccl2* and *Tnf,* in the ischemic penumbra 1 d and 3 d after tMCAO. n = 5 mice per group. *P < 0.05 versus the sham group, **P < 0.01 versus the sham group, ^#^*P* < 0.05 versus the WT group, ^##^*P* < 0.01 versus the WT group, one-way ANOVA with Bonferroni post hoc test. **b** Immunostaining for TMEM119 (green) and Iba1 (red) and 3D-reconstructed images of TMEM119^+^Iba1^+^ microglia 1 d and 3 d after tMCAO. Scale bar: 50 µm. *n* = 5 mice per group. *P < 0.05 versus the WT group, **P < 0.01 versus the WT group, unpaired Student’s t test. **c** Quantification of Iba1 MFI. **d** Quantification of average branch length, branch numbers and junctions of 3D-reconstructed microglia in the peri-infarct region. **e** One day after tMCAO, microglia sorted based on the CD45^int^ and CD11b^+^ strategy were subjected to PCR array analysis. **f** FACS analysis of IL-1β, TNF-α and IL-6 expression in microglia 1 d after tMCAO. MFI was quantified. *n* = 5 mice per group. ***P < 0.001 versus the sham group, ^##^*P* < 0.01 versus the WT group, one-way ANOVA with Bonferroni post hoc test

Properdin has emerged as a key factor that participates in various inflammatory diseases [21, 22, 27]. Therefore, we investigated the impact of properdin on brain inflammation. qPCR assessment of classical inflammatory gene expression confirmed that *Cfp*^−/−^ mice exhibited downregulated mRNA levels of *Il1b*, *Il6, Nos2*, *Ccl2* and *Tnf* compared to WT mice 1 d and 3 d after tMCAO, and the differences in *Il1b* and *Tnf* expression were particularly notable. Concomitantly, the expression levels of anti-inflammatory factors, including *Il4* and *Il10,* were partially upregulated in *Cfp*^−/−^ mice (Fig. 2a). Given the role of microglia as key regulators of brain inflammation, we then evaluated microglial activation by assessing their morphology and Iba1 staining fluorescence intensity. Immunofluorescence staining showed significantly decreased Iba1 staining in the microglia of *Cfp*^−/−^ mice (Fig. 2b, c). We reconstructed three-dimensional microglial morphologies based on the images of Iba1 and TMEM119 dual-labeled microglia. Significantly, microglia from *Cfp*^−/−^ mice had longer branch lengths, more branches, and branch junctions than those from WT mice 1 d and 3 d after tMCAO, indicating that the microglia in the ischemic brains of *Cfp*^−/−^ mice were more ramified and less reactive (Fig. 2b and d). These data indicated that properdin promotes the activation of microglia during ischemic stroke, thereby exacerbating the inflammatory response in the injured brain.

To elucidate the role of properdin in regulating microglial functions in vivo, we assessed the gene expression profiles of microglia that were sorted from ischemic hemispheres of *Cfp*^−/−^ mice and WT mice 1 d after tMCAO using mRNA microarray analysis (Fig. 2e). Overall, the expression of proinflammatory cytokines, receptors and chemokines (*Cx3cl1, Il0ra, Il0rb, Il1a, Spp1*, etc.) was decreased in microglia from *Cfp*^−/−^ mice compared to those from WT mice. After quantifying the microarray analysis results, the expression levels of *Ilb* and *Tnf* were significantly decreased in microglia sorted from *Cfp*^−/−^ mice (Additional file 1: Figure S4a). We further examined the protein levels of IL-1β, TNF-α and IL-6 in microglia from ischemic brain hemispheres using flow cytometry. One day and 3 d after tMCAO, the levels of IL-1β and TNF-α were obviously decreased in microglia from *Cfp*^−/−^ mice in comparison to those from WT mice, while no significant differences were observed in the microglial IL-6 protein levels between *Cfp*^−/−^ mice and WT mice (Fig. 2f and Additional file 1: Figure S4b). Altogether, the in vivo data show that knockout of *Cfp* can alleviate excessive inflammatory responses in microglia after ischemia.

### Recombinant mouse properdin (rmProperdin) activates microglia and induces microglia-potentiated neuronal death in vitro

**Fig. 3:**
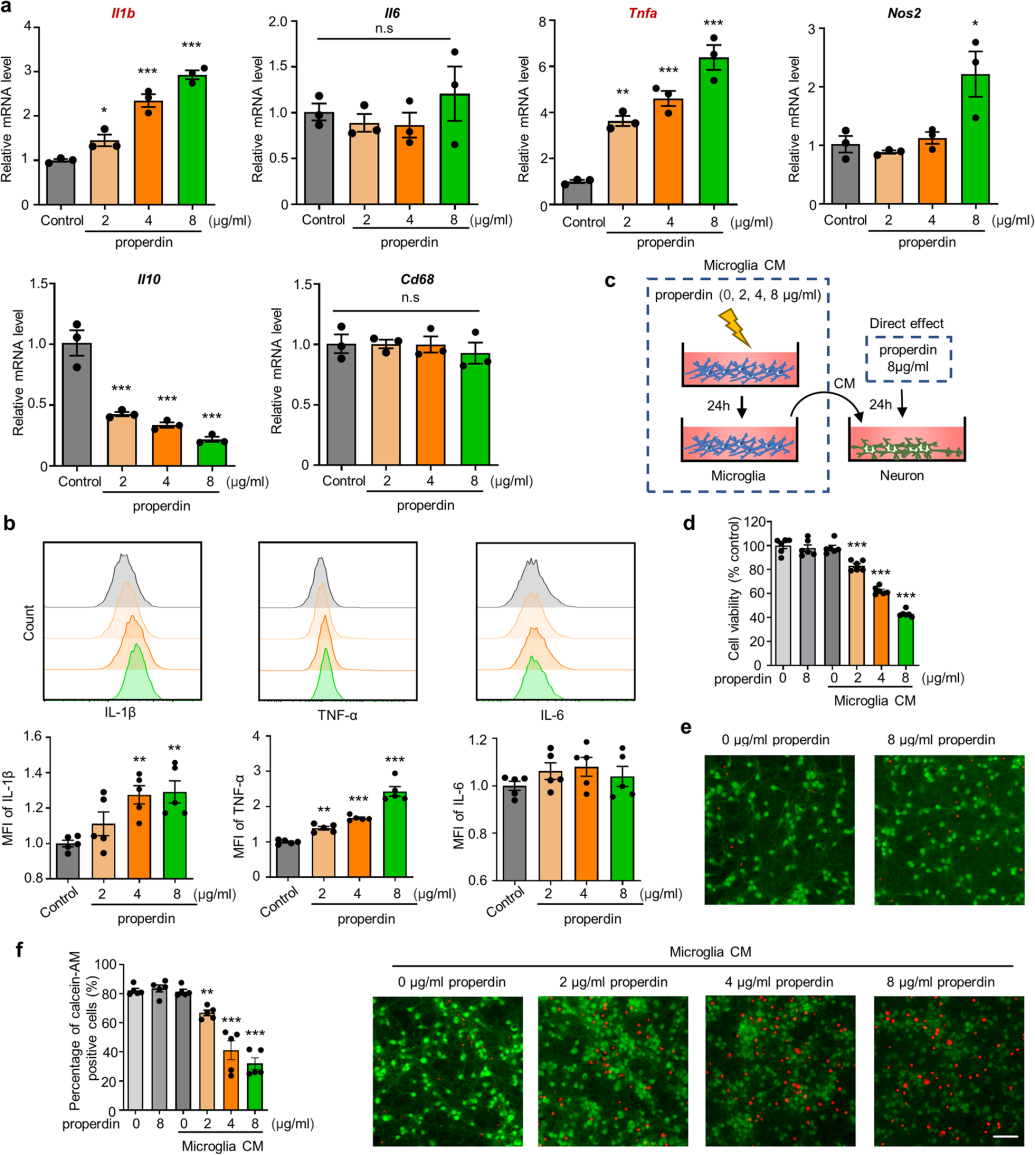
rmProperdin activates microglia and induces microglia-potentiated neuronal death in vitro. **a** The mRNA expression of inflammatory factors was measured in primary microglia treated with 2 µg/ml, 4 µg/ml and 8 µg/ml rmProperdin versus control microglia. *n* = 3 per group, *P < 0.05 versus the control group, **P < 0.01 versus the control group, ***P < 0.001 versus the control group, n.s, no significance (*P* > 0.05), one-way ANOVA with Bonferroni post hoc test. **b** FACS analysis of IL-1β, TNF-α and IL-6 expression in primary microglia treated with rmProperdin for 24 h. MFI was quantified. *n* = 3 per group, **P < 0.01 versus the control group, ***P < 0.001 versus the control group, one-way ANOVA with Bonferroni post hoc test. **c** Experimental design for the analysis of neuronal viability after treatment with rmProperdin or CM from rmProperdin-treated microglia. **d** Neuronal viability was assessed by CCK8 assay. *n* = 6 per group, ***P < 0.001 versus the control group, one-way ANOVA with Bonferroni post hoc test. **e** Analysis of neuronal death using calcein-AM (green)/PI (red) double staining. Scale bar: 50 µm. **f** Calcein-AM-positive primary cortical neurons were quantified as percentages of total cells. *n* = 5 per group, **P < 0.01 versus the control group, ***P < 0.001 versus the control group, one-way ANOVA with Bonferroni post hoc test

To further investigate the mechanism by which properdin induces microglial activation, we first stimulated primary microglia with eukaryotic recombinant mouse properdin (rmProperdin) in vitro to mimic the in vivo effects of properdin on microglia. Direct stimulation with rmProperdin did not induce microglial death, even at a high concentration (Additional file 1: Figure S5a–c). The expression of a battery of inflammatory cytokines was evaluated by qPCR analysis, and the results indicated that rmProperdin could activate primary microglia in vitro, as revealed by the increased expression levels of *Il1b* and *Tnf* as well as the decreased levels of *Il10* in rmProperdin-treated primary microglia. *Il6* and *CD68* (phagocytic marker) gene expression levels showed no significant changes when microglia were treated with different concentrations of rmProperdin (2, 4, or 8 µg/ml), while a high concentration of rmProperdin (8 µg/ml rmProperdin) increased the levels of *Nos2* in microglia (Fig. 3a). To confirm these results, we further used flow cytometry to determine the protein levels of IL-1β, TNF-α and IL-6. Similar to the in vivo results, the levels of IL-1β and TNF-α were increased in rmProperdin-treated microglia, whereas rmProperdin had a negligible effect on IL-6 production by microglia (Fig. 3b).

Given that excessive microglial inflammatory insult can cause neuronal death, we investigated whether properdin can induce microglial hyperactivation, contributing to neuronal death. We stimulated primary microglia with rmProperdin (2, 4, or 8 µg/ml) or PBS for 24 h, collected the conditioned media (CM) from the microglia, and stimulated primary neurons with this CM (Fig. 3c). rmProperdin-treated microglial CM caused neuronal death in a dose-dependent manner, as shown by the decrease in neuronal viability. However, the CM from PBS-treated microglia and direct exposure to 8 µg/ml rmProperdin had no effect on primary neurons (Fig. 3d–f). Thus, the in vitro stimulation of primary microglia with rmProperdin can induce microglial activation, similar to what occurs in vivo, ultimately causing damage to neurons.

### Molecular patterning of rmProperdin-treated primary microglia

**Fig. 4:**
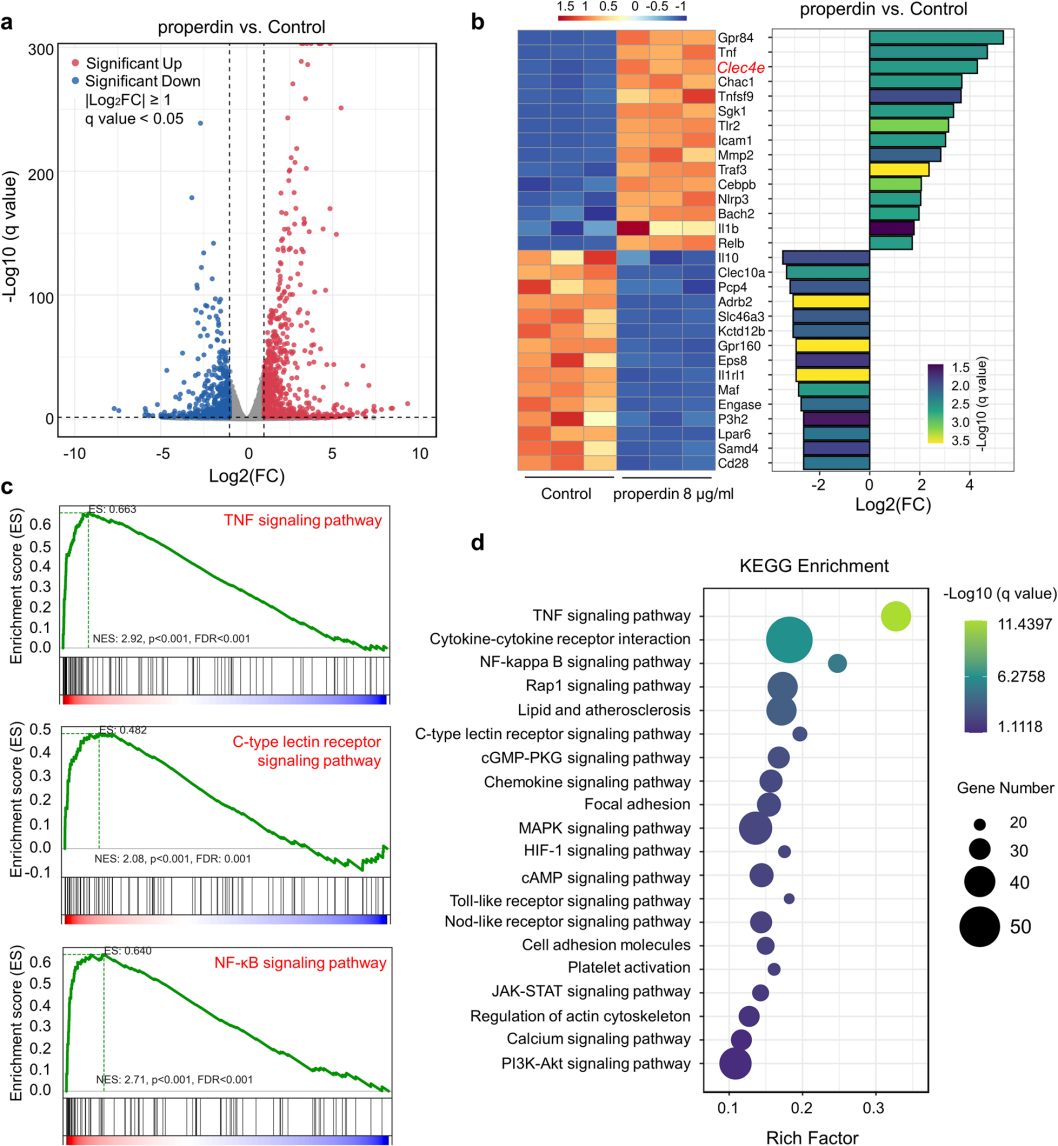
Molecular patterning of rmProperdin-treated primary microglia. **a** Volcano plot showing the upregulated and downregulated DEGs (∣log2FC∣ > 1, FDR < 0.05) between 8 µg/ml rmProperdin-treated primary microglia and control microglia. **b** Heatmap of genes differentially expressed between rmProperdin-treated microglia and unstimulated microglia. **c** GSEA plots showing that the significantly enriched gene sets positively correlated with rmProperdin-treated microglia were “TNF signaling pathway”, “NF-kappa B signaling pathway” and “C-type lectin receptor signaling pathway”. **d** Scatter plot of KEGG pathway enrichment analysis of DEGs in microglia treated with rmProperdin or not

Having confirmed that rmProperdin could activate microglia in vitro, we then collected primary microglia that were treated with 8 µg/ml rmProperdin for 3 h and performed bulk RNAseq analysis, with unstimulated microglia serving as controls. Principal component analysis (PCA) showed differences between rmProperdin-treated microglia and control microglia (Additional file 1: Figure S6a). Among the 1573 differentially expressed genes (DEGs), 744 were upregulated and 829 were downregulated between rmProperdin-treated microglia and control microglia (Fig. 4a). After stimulation with rmProperdin, microglial genes related to inflammatory processes, such as *Tnf*, *Icam1*, *Mmp2*, *Cebpb*, *Nlrp3*, *Ilb* and *Relb*, were upregulated. Genes that are associated with PRRs, such as *Clec4e* and *Tlr2*, were also upregulated (Fig. 4b). Notably, among the genes with the most significant increases in expression, *Cebpb*-encoded C/EBPβ is the downstream of the *Clec4e*-encoded receptor Mincle and mediates a positive feedback loop by inducing Mincle expression [28]. The results suggest that the Mincle pathway might play an important role in properdin-induced microglial activation. Next, we used KEGG pathway enrichment analysis and GSEA to further analyze the DEGs and found that the TNF signaling pathway, NF-κB signaling pathway and C-type lectin receptor signaling pathway were highly enriched in microglia stimulated with properdin (Fig. 4c, d and Additional file 1: Figure S6b). GO analysis also confirmed the enrichment of the inflammatory response in rmProperdin-stimulated microglia (Additional file 1: Figure S6c, d).

### Properdin binds to and activates the Mincle receptor on microglia

**Fig. 5:**
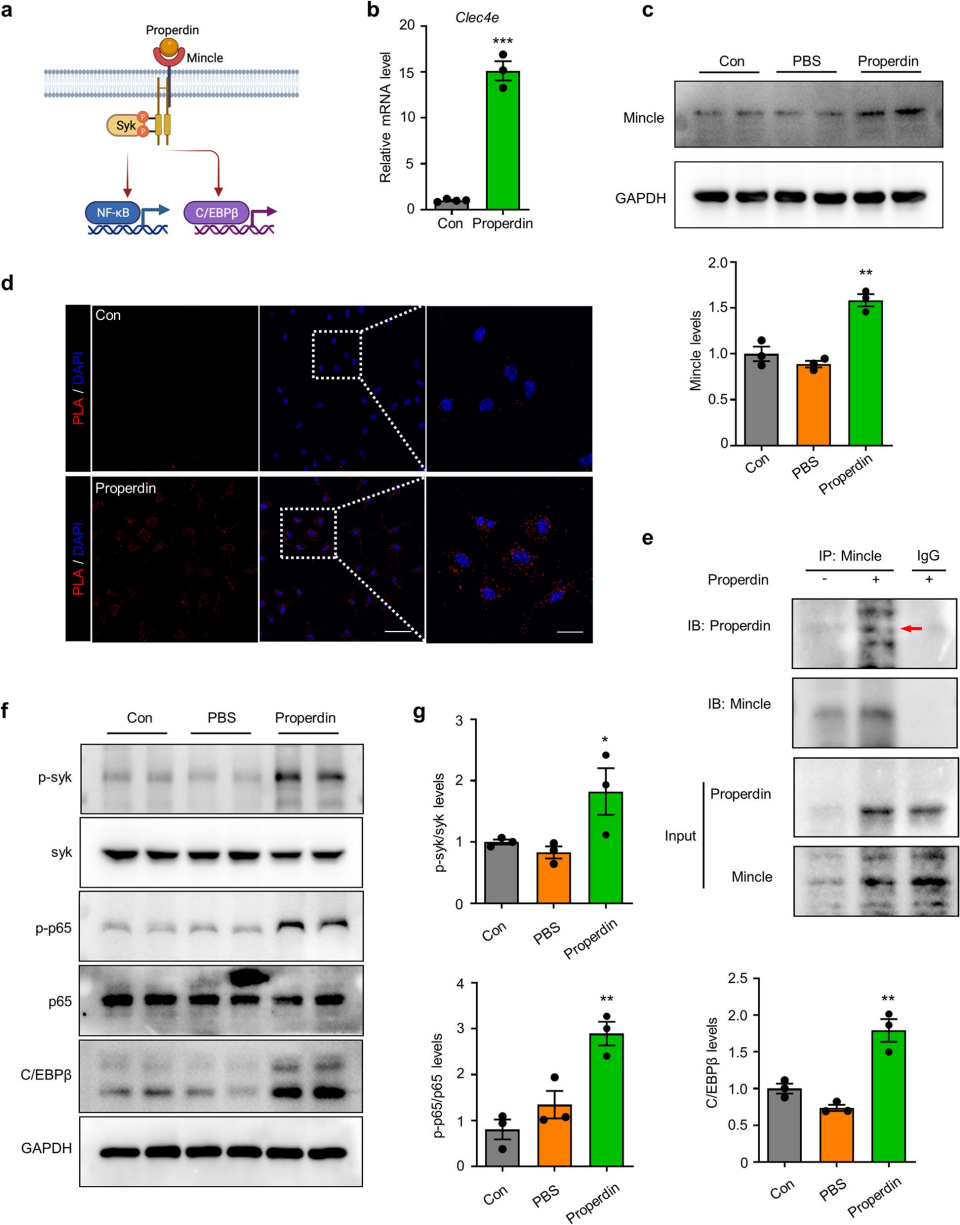
Properdin binds to and activates the Mincle receptor on microglia. **a** Description of Mincle receptor-mediated downstream signaling pathways. **b** Expression of *Clec4e* in rmProperdin-treated microglia versus control microglia was evaluated by qPCR. *n* = 3 to 4 per group, ***P < 0.001, unpaired Student’s t test. **c** Mincle receptor expression was assessed by Western blotting. *n* = 3 per group, **P < 0.01 versus the control group, one-way ANOVA with Bonferroni post hoc test. **d** Representative images of the Duolink PLA assay showing the PLA signals elicited by the interaction of rmProperdin and the Mincle receptor on primary microglia. **e** After immunoprecipitation with anti-Mincle, lysates of primary microglia treated with or without 8 µg/ml rmProperdin were immunoblotted with anti-properdin and anti-Mincle antibodies. Mouse IgG served as a control. **f**, **g** Western blotting analysis of p-Syk, p-p65, and C/EBPβ expression in rmProperdin-treated microglia versus control microglia. Quantification of the p-Syk/Syk ratio, p-p65/p65 ratio and C/EBPβ expression was normalized to the expression of the housekeeping gene GAPDH. *n* = 3 per group, *P < 0.05, **P < 0.01 versus the control group, one-way ANOVA with Bonferroni post hoc test

The PRR Mincle often recognizes glucose- or mannose-containing ligands, and coincidentally, properdin harbors glycosylation and mannosylation sites [29, 30]. Based on the RNAseq analysis described above, we hypothesized that properdin could bind to the receptor Mincle on microglia to exert its effect (Fig. 5a). Consistent with the RNAseq analysis, qPCR confirmed that *Clec4e* expression was upregulated in rmProperdin-stimulated microglia (Fig. 5b), and the protein levels of Mincle were increased in microglia treated with properdin (Fig. 5c). We next explored whether Mincle could recognize and capture the properdin protein. A Duolink proximity ligation assay (PLA) was conducted on rmProperdin-treated microglia, and the interaction of Mincle and rmProperdin was examined by visualizing the PLA signals (Fig. 5d). A co-immunoprecipitation assay also validated the Mincle–properdin interaction in primary microglia (Fig. 5e). Consistently, properdin also bound to Mincle in the ischemic brain tissue, further confirming the interaction in vivo (Additional file 1: Figure S7). Spleen tyrosine kinase (Syk), NF-κB and C/EBPβ are well-recognized downstream effectors of Mincle [28, 31, 32]. The administration of rmProperdin activated Syk and NF-κB as well as increased the levels of C/EBPβ in primary microglia compared to unstimulated and PBS-treated microglia (Fig. 5f, g). Taken together, these results demonstrated that properdin could bind to the Mincle receptor on microglia and further activate the downstream pathway of Mincle.

### *Clec4e* silencing suppresses the proinflammatory effect of properdin on microglia

**Fig. 6:**
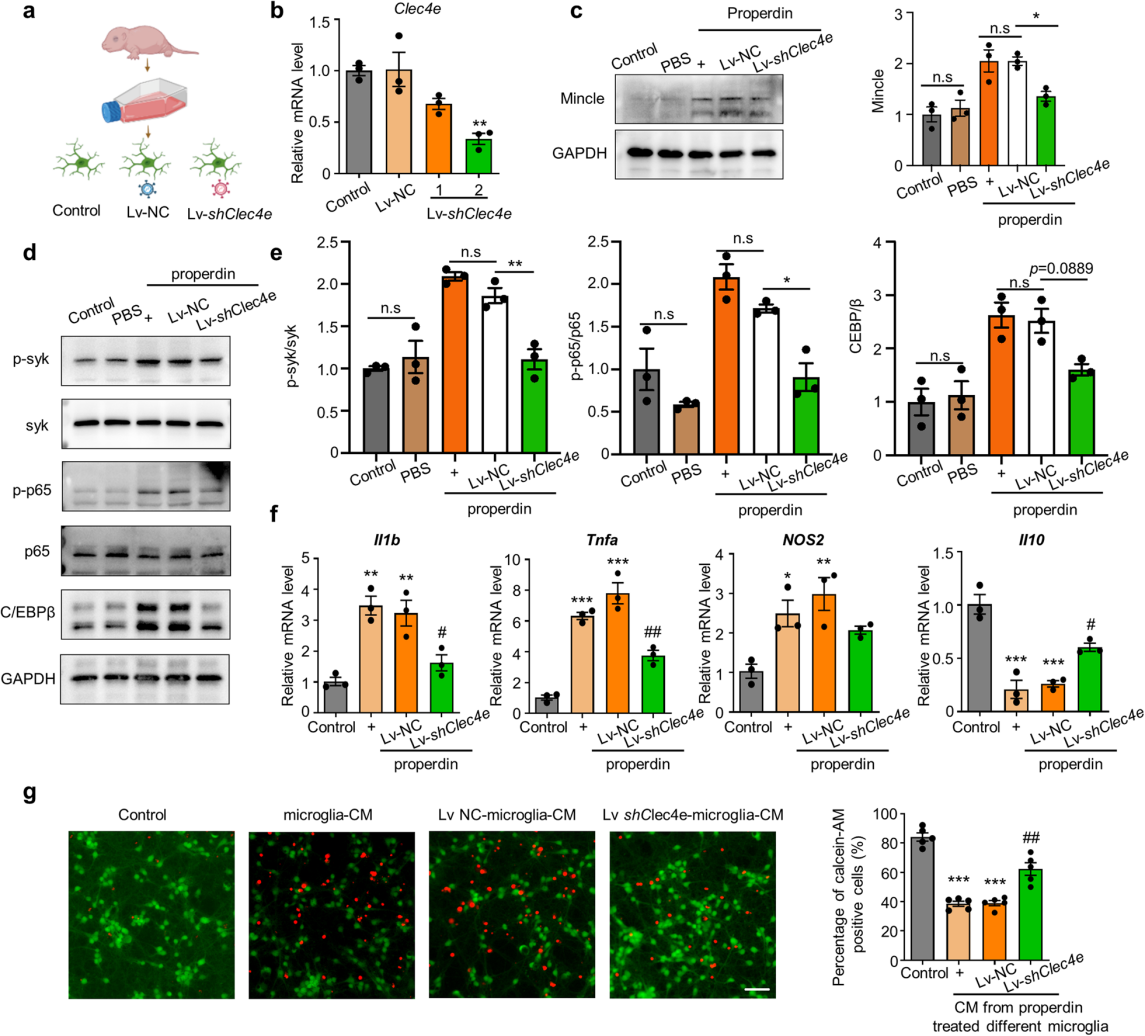
*Clec4e* silencing suppresses the proinflammatory effect of properdin on microglia. **a** Experimental design for *Clec4e* silencing in primary microglia. **b** The transfection efficiency of lentivirus in microglia was determined by qPCR. *n* = 3 per group, **P < 0.01 versus the Lv-NC group, one-way ANOVA with Bonferroni post hoc test. **c** Western blotting analysis of Mincle expression after *Clec4e* silencing. *n* = 3 per group, *P < 0.05 versus the Lv-NC + properdin group, one-way ANOVA with Bonferroni post hoc test. **d** Protein expression of p-Syk, p-p65 and C/EBPβ in microglia after silencing *Clec4e*. **e** Quantification of the p-Syk/Syk ratio, p-p65/p65 ratio and C/EBPβ expression was normalized to GAPDH expression. *n* = 3 per group, *P < 0.05 versus the Lv-NC + properdin group, **P < 0.01 versus the Lv-NC + properdin group, one-way ANOVA with Bonferroni post hoc test. **f** mRNA levels of *Il1b*, *Tnf*, *Nos2* and *Il10* in microglia after *Clec4e* knockdown. *n* = 3 per group, *P < 0.05 versus the control group, **P < 0.01 versus the control group, ***P < 0.001 versus the control group, ^#^*P* < 0.05 versus the Lv-NC + properdin group, ^##^*P* < 0.01 versus the Lv-NC + properdin group, one-way ANOVA with Bonferroni post hoc test. **g** Images of calcein-AM/PI staining showing the level of neuronal death. The data were quantified as the percentage of calcein-AM-positive cells. *n* = 5 per group, ***P < 0.001 versus the control group, ^##^*P* < 0.01 versus the Lv-NC + CM group, one-way ANOVA with Bonferroni post hoc test

To better elucidate that the functional role of properdin in activating microglia is mainly mediated by the direct interaction between properdin and Mincle, we generated primary microglia with *Clec4e*-knockdown using lentivirus shRNA (Fig. 6a–c). Analysis of the Mincle-mediated signaling pathway revealed that *Clec4e* silencing suppressed the rmProperdin-induced activation of downstream effectors, as indicated by the lower levels of p-Syk, p-p65 and C/EBPβ in *Clec4e*-knockdown microglia than in control microglia (Fig. 6d, e). Consistently, even after treatment with rmProperdin, *Clec4e*-knockdown microglia exhibited downregulation of proinflammatory gene expression levels (*Il1b* and *Tnf*) and upregulated expression of the anti-inflammatory gene *Il10* compared to control microglia (Fig. 6f). To investigate whether *Clec4e* silencing could alleviate the properdin-induced neurotoxicity of microglia, we conducted Calcein-AM and PI staining to evaluate neuronal viability. As shown in Fig. 6g, CM from rmProperdin-stimulated microglia caused neuronal death, while knocking down *Clec4e* in microglia weakened the effect of properdin. Overall, these data show that disrupting the interaction of properdin and Mincle by silencing *Clec4e* could reverse the effect of properdin on microglia.

### *Cfp* deficiency in macrophages and neutrophils attenuates inflammation and protects against ischemic brain injury

**Fig. 7:**
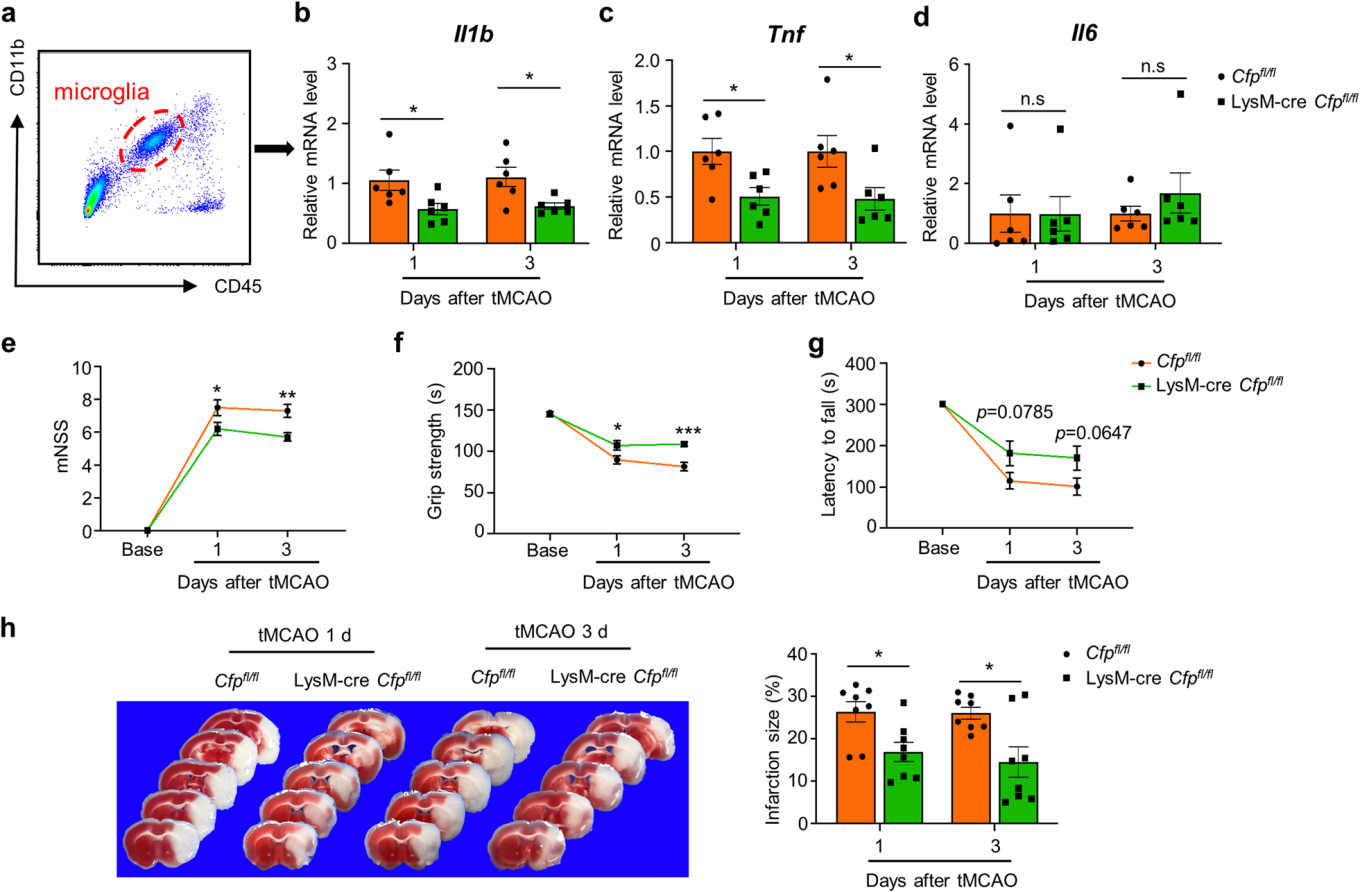
*Cfp* deficiency in macrophages and neutrophils attenuates inflammation and protects against ischemic brain injury. **a** Gating strategy for microglia based on CD45^int^ and CD11b^+^. **b-d** qPCR assessment of *Il1b*, *Tnf* and *Il6* expression levels in microglia isolated from mice 1 d and 3 d after tMCAO. *n* = 6 mice per group, *P < 0.05 versus the *Cfp*^fl/fl^ group, unpaired Student’s t test. **e**–**g** Evaluation of neurological function between LysM-cre *Cfp*^fl/fl^ and *Cfp*^fl/fl^ mice according to mNSS score (**e**), grip strength (**f**) and rotarod test (**g**) at 1 d and 3 d after tMCAO. *n* = 10 mice per group, *P < 0.05 versus the *Cfp*^fl/fl^ group, **P < 0.01 versus the *Cfp*^fl/fl^ group, ***P < 0.001 versus the *Cfp*^fl/fl^ group, two-way ANOVA with Sidak's multiple comparisons test. **h** Representative images and quantification of the infarct area as assessed by TTC staining 1 d and 3 d after tMCAO. *n* = 8 mice per group, *P < 0.05 versus the *Cfp*^fl/fl^ group, unpaired Student’s *t* test

Existing data suggest that properdin in the brain is mainly produced by macrophages and neutrophils. To further confirm whether microglial activation in ischemic brains is mediated by properdin from macrophages and neutrophils, we generated LysM-cre *Cfp*^fl/fl^ mice (*Cfp*-cKO mice) to deplete *Cfp* in macrophages and neutrophils (Additional file 1: Figure S8a, b). Microglia were isolated from *Cfp*-cKO mice and WT littermates (*Cfp*^fl/fl^ mice) 1 d and 3 d after tMCAO to evaluate microglial activation. The results showed that *Il1b* and *Tnf* expression levels in microglia were downregulated in *Cfp*-cKO mice (Fig. 7a–d). Furthermore, we assessed ischemic brain injury in *Cfp*-cKO mice subjected to tMCAO, and *Cfp*-cKO mice had similar rCBF to WT mice (Additional file 1: Figure S8c). Similar to *Cfp*^−/−^ mice, *Cfp*-cKO mice had improved neurological function, greater grip strength, and more time spent on the rotarod cylinder (Fig. 7e–g). Accordingly, *Cfp*-cKO mice exhibited a decreased infarct size (Fig. 7h). These results suggest that the depletion of properdin in macrophages and neutrophils suppresses microglial activation, thereby alleviating ischemic brain injury.

## Discussion

**Fig. 8:**
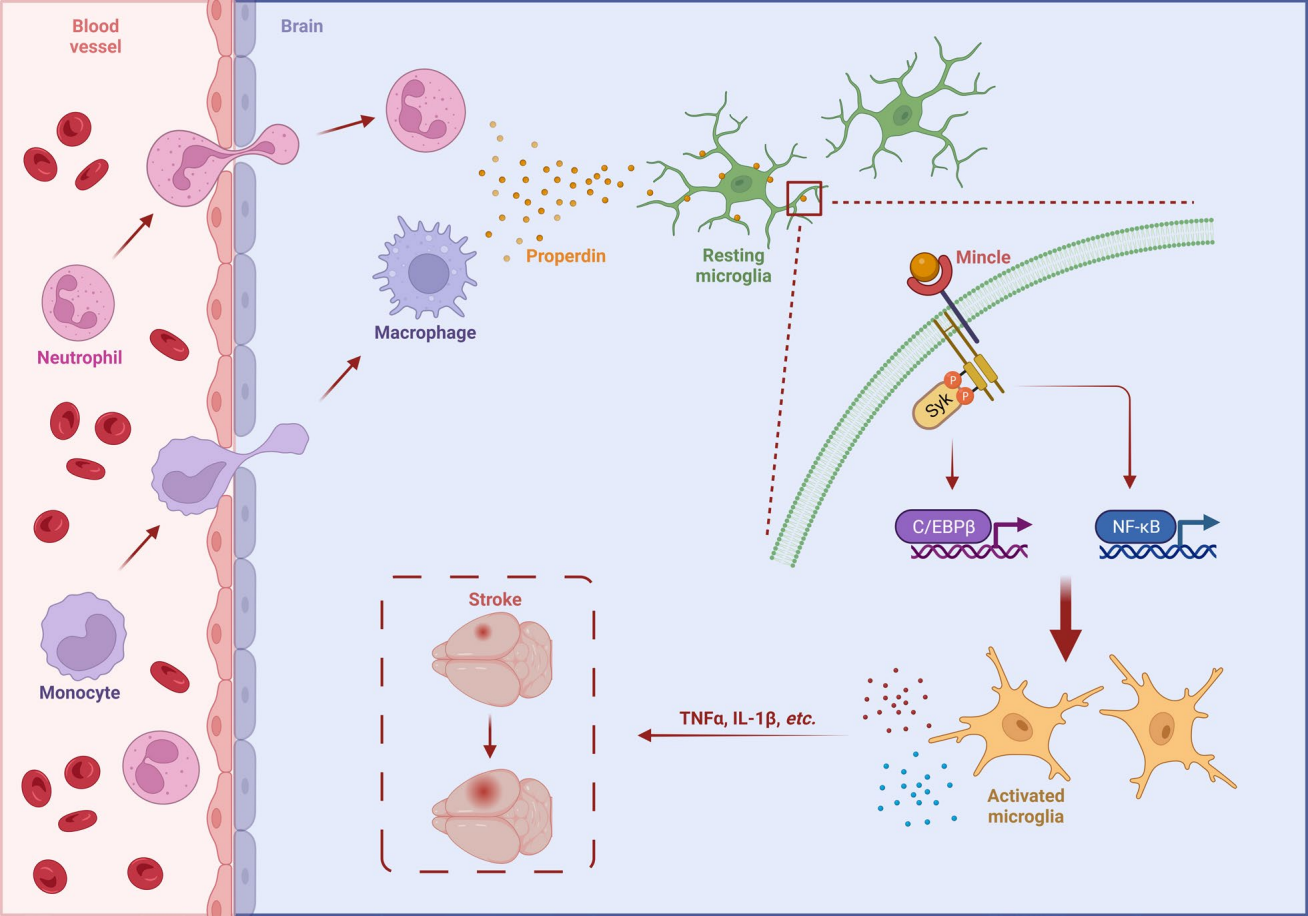
Properdin released by myeloid cells exerts proinflammatory effects by directly binding to the microglial Mincle receptor and activating the downstream transcription factors NF-κB and C/EBPβ

In the present study, we provide the first evidence that increased properdin expression in the infarcted hemisphere plays a crucial role in exacerbating ischemic brain injury. Our findings shed new light on the mechanism underlying microglia-mediated inflammation after ischemic stroke. Our results suggest that (1) infiltrating myeloid cells, mainly neutrophils and macrophages, are the source of properdin in the brain after ischemic stroke; (2) global and conditional (myeloid-specific) knockout of properdin attenuates microglial overactivation and microglia-induced inflammation, leading to protection against ischemic brain injury in an experimental mouse model of stroke; (3) stimulation of primary microglia with recombinant properdin in vitro significantly augments inflammation; (4) properdin promotes neuroinflammation by directly binding to microglial Mincle receptors and then activating downstream signaling pathways; and (5) Mincle knockdown inhibits the properdin-induced proinflammatory response. Altogether, these findings broaden the understanding of how neutrophils and macrophages interact with resident cells in the brain to determine stroke outcomes: neutrophils and macrophages secrete properdin, which activates the Mincle/Syk signaling pathway to promote microglial proinflammatory responses to exacerbate brain injury (Fig. 8). Therefore, properdin may be a potential therapeutic target in ischemic stroke.

After cerebral ischemia, peripheral immune cells sequentially infiltrate into the ischemic brain and participate in the process of ischemic brain damage [33, 34]. Neutrophils are the first immune cells to be attracted to the brain, and their numbers significantly increase 3 h after ischemia, peak at 24 h, and decrease within 7 days [35]. Monocytes are the precursors of macrophages that can differentiate into macrophages after infiltrating ischemic brain tissue. Unlike neutrophils, the number of macrophages peaks 3–7 d after ischemic injury and can persist for one month or more [36]. At the beginning of this study, single-cell transcriptomics revealed that neutrophils and macrophages were the main sources of properdin. Meanwhile, the results of properdin expression at different timepoints during the acute phase after cerebral ischemia seemed to be consistent with the duration of these two cell types remaining in the brain. That is, with the increase in neutrophil and macrophage numbers in the brain, the expression of properdin also increased. Moreover, the expression of properdin was still higher than that in the sham group on day 7, which might be related to the large number of macrophages in the brain. Although there has been a debate about the temporal dynamics of the infiltration of myeloid cells [35], it is widely acknowledged that neutrophils and macrophages infiltrate the brain parenchyma at the very early stage of ischemic stroke, suggesting their role in interacting with resident microglia to initiate or strengthen post-stroke neuroinflammation, which is also closely related to our main findings in this study. In addition, lymphocytes are also considered a leukocyte subpopulation that causes high degrees of secondary inflammatory brain injury [24, 37]. In tMCAO models, lymphocyte infiltration does not reach its peak until 5 days after ischemic onset [38]. Given that we focused on the effect of properdin in acute ischemic stroke (within 1–3 days post-ischemia), lymphocytes were not explored in this study.

Importantly, although the scRNA-seq results suggested that microglia did not express properdin, microglia are the resident macrophages in the brain and are phenotypically similar to monocyte-derived macrophages. Therefore, we stained brain sections to observe whether microglia also express properdin. Interestingly, the results showed that properdin colocalized with Iba1^+^TMEM119^−^ macrophages, whereas colocalization with TMEM119^+^ microglia was barely observed. Our findings are consistent with those of Werner et al., who suggested that there are clear differences between macrophages and microglia in the expression of a series of genes related to immune response processes, including properdin [39].

Properdin is a plasma glycoprotein that is well-known for its function as a positive regulator of the alternative complement pathway [40]. Several studies have shown that properdin can perform functions that are not related to the complement pathway in a variety of disease processes. For example, it was reported that high properdin expression in breast cancer induces apoptosis by upregulating the proapoptotic transcription factor DDIT3, which is related to the endoplasmic reticulum stress response [41]. Anti-properdin monoclonal antibodies are believed to reduce renal tubular cell apoptosis after renal ischemia‒reperfusion and reduce airway inflammation in asthma model mice [22, 27]. Additionally, properdin was found to alter the pro- and anti-inflammatory responses during the pathogenesis of tuberculosis [21]. However, the role of properdin in ischemic stroke remains unclear. In in vivo experiments, we confirmed that properdin knockout or myeloid cell-conditional properdin knockout improved stroke outcomes, which were associated with the suppression of excessive neuroinflammation, suggesting that inhibition of properdin is beneficial for ischemic stroke recovery.

One unanswered question is which cells properdin acts on to affect neuroinflammation after stroke. Microglia are the main initiators of neuroinflammation in the CNS, and communication between microglia and peripheral immune cells has been the focus of research on diseases that affect the CNS [8, 42–44]. We have previously demonstrated that double-negative T cell numbers are increased in the brain and promote inflammatory microglial activation by producing TNF-α [24]. Additionally, we and others confirmed that invasive CD4^+^ T cells can stimulate the polarization of microglia towards a proinflammatory phenotype [45], and Tregs, which are a minor subpopulation of CD4^+^ T cells, produce osteopontin to facilitate microglial polarization toward the anti-inflammatory phenotype [37]. Therefore, we speculate that myeloid cell-derived properdin can also affect neuroinflammation by acting on microglia. We first observed and compared the morphology of microglia from *Cfp*^*−/−*^ and WT mice subjected to tMCAO and found that microglial priming was reduced in *Cfp*^*−/−*^ mice. Then, we confirmed that properdin not only induced the mRNA and protein expression of IL-1β and TNF-α in primary microglia, but also promoted the production of these proinflammatory cytokines in the brains of tMCAO model mice. However, properdin had no effect on the production of IL-6. IL-1β and TNF-α have been implicated in ischemic brain injury. IL-1β levels are increased early in peri-infarct microglia, and a localized striatal injection of IL-1β exacerbates ischemic brain damage [46, 47]. Microglia are thought to be the main source of TNF-α in the brain, and secreted TNF-α can directly act on TNF receptor 1 in neurons to cause cell death [48, 49]. In addition to IL-1β and TNF-α, IL-6 is also a pleiotropic cytokine that is involved in stroke, but its function is still controversial. Although accumulating evidence suggests that the inhibition of IL-6 can exert a protective effect on the brain, studies by Ali et al. showed that ischemia-induced IL-6 was an endogenous cytokine that exerted neuroprotective effects against NMDA receptor-mediated injury [50, 51]. Notably, microglia are involved in phagocytosis and chemotaxis in addition to regulating neuroinflammation [52]. However, in this study, the presence and absence of properdin did not affect these two functions of microglia. This is consistent with the current view that properdin is a critical inflammatory modulator in many diseases [22, 53].

To further elucidate the detailed mechanism by which properdin modulates the proinflammatory function of microglia, we compared the transcriptomes of microglia treated with or without rmProperdin. Both GO analysis and KEGG pathway enrichment analyses indicated that the inflammatory response of microglia was significantly changed after properdin administration. We noticed that among the 744 upregulated DEGs, *Clec4e* expression was elevated by more than 20 times. The Mincle protein, which is encoded by *Clec4e*, is an important CLR that is believed to play a distinct role in driving inflammatory factor production [54, 55]. Moreover, emerging evidence has demonstrated that Mincle is involved in the activation of microglia after traumatic brain injury and subarachnoid hemorrhage. Blocking microglial Mincle or its downstream targets attenuates neurofunctional damage and neuroinflammation [56, 57]. It has been reported that Mincle can serve as a PRR and is directly activated by DAMPs [58]. As mentioned above, properdin is considered a DAMP that activates NK cells and lymphoid cells [16], indicating that properdin may exert its proinflammatory effects by binding to microglial Mincle. As expected, the results of in situ proximity ligation assays and Co-IP confirmed that there is indeed a direct interaction between properdin and Mincle in microglia.

Since Mincle induced inflammatory responses via the direct downstream effector Syk, the potential role of properdin in this signaling pathway was further explored. NF-κB is recognized as a key downstream transcription factor that is regulated by Mincle/Syk, which controls the expression of proinflammatory cytokines in microglia [56, 59]. Moreover, it should be noted that *Cebpb* showed a significant increase among the upregulated DEGs, and *Cebpb*-encoded protein C/EBPβ was found to be a central hub in Mincle expression and inflammatory gene induction [28]. Our study revealed that Syk and NF-κB were activated and that C/EBPβ expression was increased in properdin-treated microglia. In this study, we focused on Mincle/Syk and the two downstream transcription factors and therefore did not explore the intermediate link between Syk and NF-κB or C/EBPβ. Finally, considering the lack of Mincle-specific inhibitors, a lentivirus-mediated gene silencing was chosen to decrease Mincle expression in this study. Mincle knockdown reversed the properdin-induced activation of downstream signaling pathways and ameliorated microglial inflammation.

There are also several limitations in this study. First, we used tMCAO model instead of permanent MCAO (pMCAO) model because the Stroke Treatment Academic Industry Roundtable (STAIR) proposed that neuroprotective agents can and should be developed in conjunction with recanalization because successful reperfusion is a necessity for neuroprotection [60–62]. Therefore, since we intended to explore the neuroprotective effect of *Cfp*-deletion, we chose tMCAO instead of pMCAO as a stroke model. However, we will still further investigate the role of *Cfp* in pMCAO in the future. Second, mRNA analysis, especially qPCR, was used in our study to evaluate the expression levels of a panel of inflammatory factors. However, it would be more beneficial to examine the protein level of these cytokines. Additionally, this study focused on the modulation of microglia-derived inflammatory factors by infiltrating myeloid cells, while neutrophils and macrophages per se also secrete various cytokines and are involved in neuroinflammation at the acute stage of stroke, which were ignored in our study. Third, even though flow cytometry allows the isolation of pure microglia, the tremendous amount of pre-processing might affect RNA and protein expression. Finally, primary microglia, which we repeatedly used in in vitro experiments, might lose many of their functions compared to cells in vivo and are more closely related to activated macrophages [63]. Therefore, we will try to employ newly developed models (e.g., iPSC-derived human microglia and in vivo neuroimmune organoid models) that mimic microglia in vivo and their interaction with brain environment in the future [64, 65].

## Conclusions

In summary, we reported for the first time that properdin-knockout was beneficial for the ischemic brain during the early phase of stroke, which was attributed to the detrimental role of properdin that promotes microglial inflammatory responses after cerebral ischemia. Mechanistically, the Mincle/Syk signaling pathway is directly targeted by properdin. Properdin plays a proinflammatory role by directly binding to the microglial Mincle receptor and activating the downstream transcription factors NF-κB and C/EBPβ. It has been reported that another Clec protein, Clec-2, could interact with its ligand, podoplanin, to promote platelet spreading [66]. Sowa et al. recently reported that MAS9, a small molecule inhibitor, could inhibit Clec-2-podoplanin and act as a novel anti-platelet compound [67]. Furthermore, Syk functions as the downstream effector of the Mincle receptor. Accordingly, Bl1002494, a novel Syk inhibitor, has been found to protect mice from ischemic brain injury [68]. Hence, compounds that disrupt the binding between properdin and Mincle or inhibit their downstream signaling may ameliorate stroke progression and become potential candidates for stroke treatment.

### Supplementary Information

**Additional file 1: Figure S1.** The examination of properdin expression using ELISA and violin plot of Cfp gene expression in various cell clusters. **Figure S2.** Properdin expression in different regions after tMCAO. **Figure S3.** Construction strategy of Cfp-/- mice. **Figure S4.** The inflammatory levels of microglia sorted from tMCAO Cfp-/- mice and WT littermates. **Figure S5.** rmProperdin did not induce primary microglial death. **Figure S6.** RNAseq analysis on rmProperdin-treated microglia and control microglia. **Figure S7.** The interaction between properdin and Mincle in the brain tissues. **Figure S8.** Construction strategy of LysM-cre Cfpfl/fl mice, and LysM-cre Cfpfl/fl had similar rCBF with Cfpfl/fl mice.

## Data Availability

The data that support the findings of this study are available from the corresponding author upon reasonable request.

## Abbreviations

BBB: Blood–brain barrier
CM: Conditioned media
DAMPs: Damage-associated molecular patterns
DEGs: Differentially expressed genes
FACS: Fluorescence-activated cell sorting
KO: Knock out
Mincle: Macrophage-inducible C-type lectin
mNSS: Modified neurological severity score
PCA: Principal component analysis
PLA: Proximity ligation assay
PRRs: Pattern recognized receptors
qPCR: Quantitative PCR
rCBF: Regional cerebral blood flow
rmProperdin: Recombinant mouse properdin
scRNA-seq: Single-cell RNA sequencing
SEM: Standard error of the mean
Syk: Spleen tyrosine kinase
tMCAO: Transient middle cerebral artery occlusion
TTC: 2,3,5-Triphenyltetrazolium chloride
WT: Wild-type
