# Supplementary material for: Infiltrating myeloid cell-derived properdin markedly promotes microglia-mediated neuroinflammation after ischemic stroke
Source: J Neuroinflammation. 2023 Nov 11;20:260. doi: 10.1186/s12974-023-02946-z (PMC10640761; doi:10.1186/s12974-023-02946-z)
Supplement: Supplementary file 1 — Additional file 1: Figure S1. The examination of properdin expression using ELISA and violin plot of Cfp gene expression in various cell clusters. Figure S2. Properdin expression in different regions after tMCAO. Figure S3. Construction strategy of Cfp-/- mice. Figure S4. The inflammatory levels of microglia sorted from tMCAO Cfp-/- mice and WT littermates. Figure S5. rmProperdin did not induce primary microglial death. Figure S6. RNAseq analysis on rmProperdin-treated microglia and control microglia. Figure S7. The interaction between properdin and Mincle in the brain tissues. Figure S8. Construction strategy of LysM-cre Cfpfl/fl mice, and LysM-cre Cfpfl/fl had similar rCBF with Cfpfl/fl mice. [file 12974_2023_2946_MOESM1_ESM.docx]

**Additional material for**

**Infiltrating myeloid cell-derived properdin markedly promotes microglia-mediated neuroinflammation after ischemic stroke**

Pin-yi Liu, Hui-qin Li, Meng-qi Dong, Xin-ya Gu, Si-yi Xu, Sheng-nan Xia, Xin-yu Bao, Yun Xu, Xiang Cao

**This file includes:**

**Figure S1 to S8**

Figure S1. The examination of properdin expression using ELISA and violin plot of *Cfp* gene expression in various cell clusters.

Figure S2. Properdin expression in different regions after tMCAO.

Figure S3. Construction strategy of *Cfp*^-/-^ mice.

Figure S4. The inflammatory levels of microglia sorted from tMCAO *Cfp*^-/-^ mice and WT littermates.

Figure S5. rmProperdin did not induce primary microglial death.

Figure S6. RNAseq analysis on rmProperdin-treated microglia and control microglia.

Figure S7. The interaction between properdin and Mincle in the brain tissues.

Figure S8. Construction strategy of LysM-cre *Cfp*^fl/fl^ mice, and LysM-cre *Cfp*^fl/fl^ had similar rCBF with *Cfp*^fl/fl^ mice.

**Additional Figures:**

**
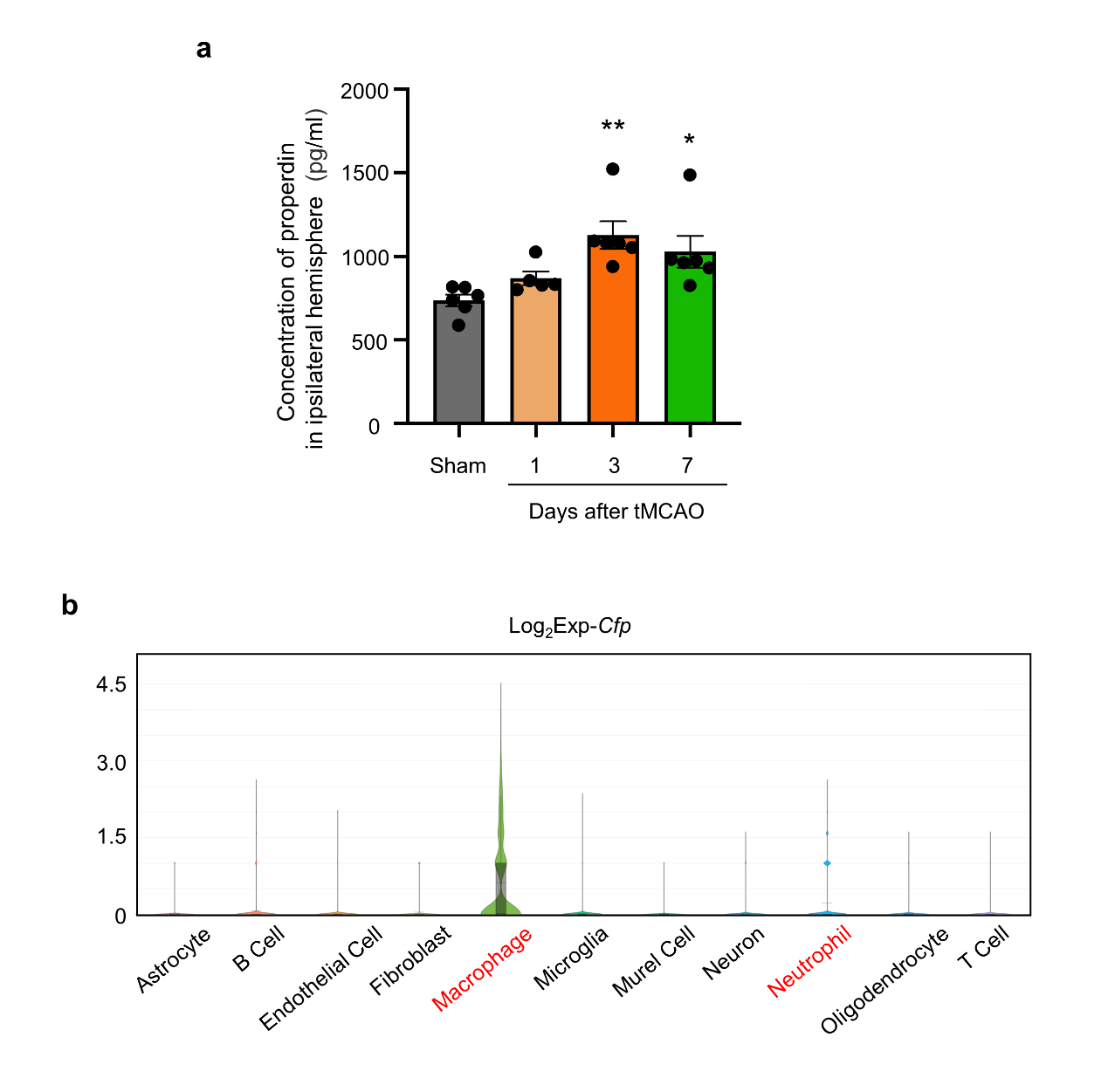
**

**Figure S1. The examination of properdin expression using ELISA and violin plot of *Cfp* gene expression in various cell clusters.**

(a) ELISA analysis of properdin concentration in the brain tissues from sham and tMCAO mice at different timepoints. n=6 in sham, 3 d and 7 d after tMCAO group and n=5 in 1 d after tMCAO group. **P*<0.05 versus the sham group, ***P*<0.01 versus the sham group, one-way ANOVA with Bonferroni post hoc test.

(b) The expression level of Cfp in various cell types in the scRNA-seq data.

**
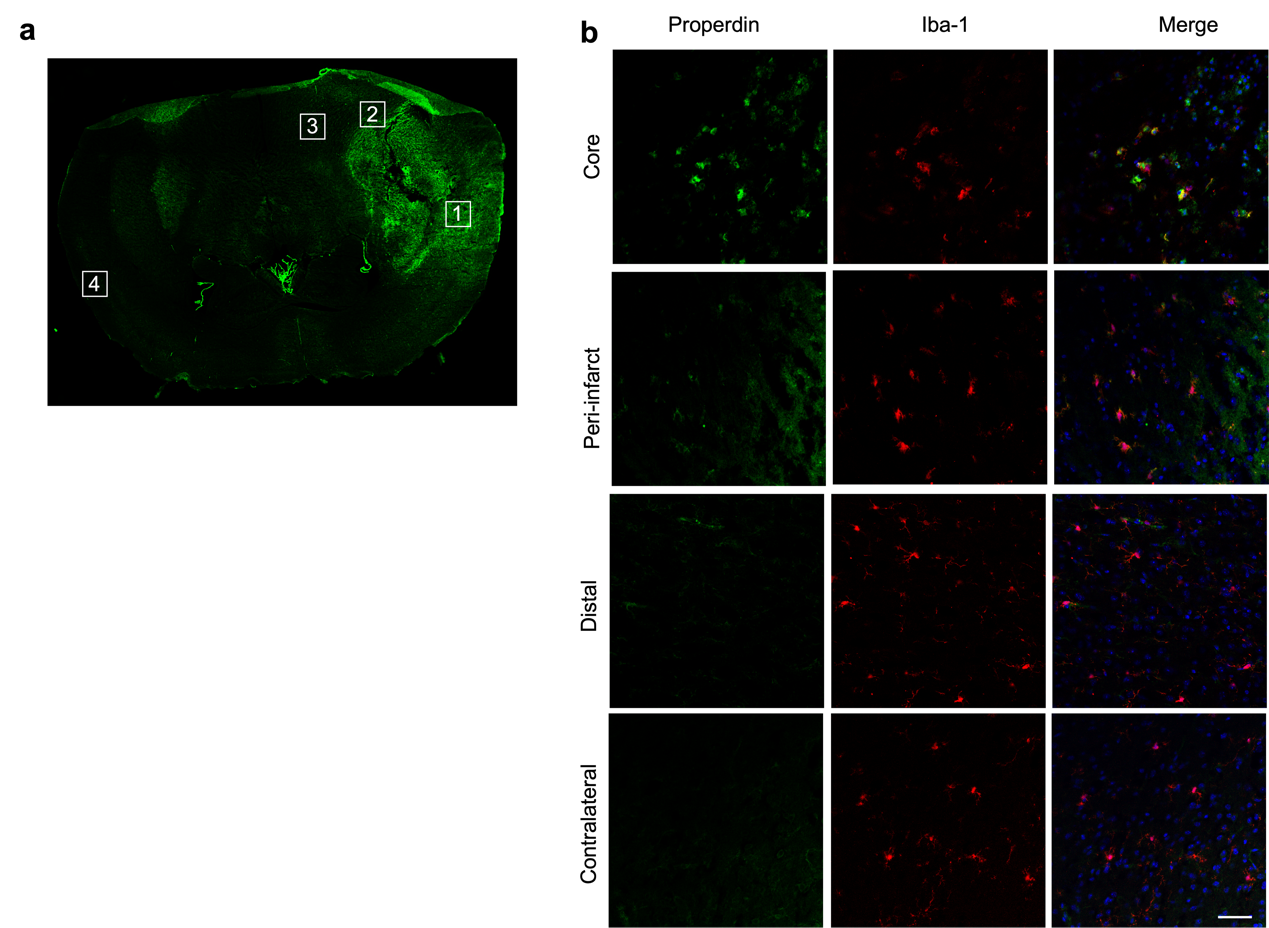
**

**Figure S2. Properdin expression in different regions after tMCAO.**

(a) Selected regions in the tMCAO brain section with properdin staining (1: core, 2: peri-infarct area, 3: distal area, 4: contralateral area).

(b) Immunostaining for properdin and Iba1 in the selected regions of C57BL/6J (B6) mice 3 d after tMCAO. Scale bars: 50 µm.

**Figure S3. Construction strategy of *Cfp*^-/-^ mice.**

(a) The gene targeting strategy to generate *Cfp*^-/-^ mice.

(b) PCR analysis for genotyping *Cfp*^-/-^ and WT mice. The homozygous mice (*Cfp*^-/-^) produced only the ~576 bp amplicon. The WT mice (*Cfp*^+/+^) produced only the ~551 bp amplicon. The heterozygous mice (*Cfp*^+/-^) produced both an ~576 bp amplicon and an ~551 bp amplicon.

**Figure S4. The inflammatory levels of microglia sorted from tMCAO *Cfp*^-/-^ mice and WT littermates.**

(a) Quantification of the mRNA microarray analysis of microglia sorted from mouse brains 1 d after tMCAO.

(b) FACS analysis of IL-1β, TNFα and IL-6 expression in microglia 3 d after tMCAO. MFI was quantified. n = 5 mice per group. **P*<0.05 versus the WT group, unpaired Student’s t test.

**Figure S5.** **rmProperdin did not induce primary microglial death.**

(a) FACS analysis of Annexin V and 7-AAD staining of primary microglia treated with or without rmProperdin.

(b) Cell viability of primary microglia was quantified. n = 3 per group, n.s, no significance (P>0.05), one-way ANOVA with Bonferroni post hoc test.

(c) LDH assay was used to assess the cytotoxicity of rmProperdin. n = 4 per group, n.s, no significance (P>0.05), one-way ANOVA with Bonferroni post hoc test.

**Figure S6**. **RNAseq analysis on rmProperdin-treated microglia and control microglia.**

(a) PCA plot of rmProperdin-treated primary microglia and control microglia.

(b) Network of KEGG pathways. Blue nodes represent DEGs, and red arrows represent enriched pathways.

(c) GO functional analysis of DEGs in biological process, molecular function and cellular component.

(d) Chord diagram of the relation between GO terms and DEGs.


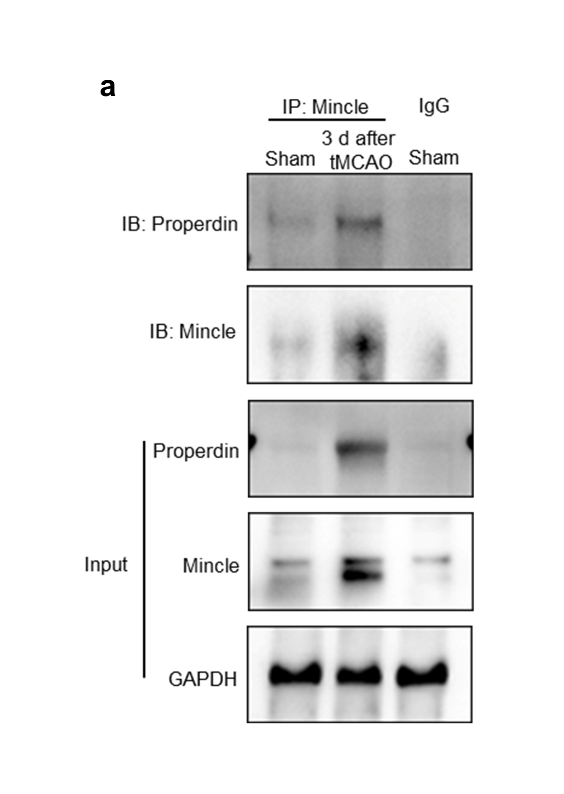


**Figure S7. The interaction between properdin and Mincle in the brain tissues.**

(a) Immunoblot analysis of properdin in the lysates (sham and tMCAO brain tissues) immunoprecipitated with an anti-Mincle antibody. Mouse IgG served as a negative control.

**Figure S8**. **Construction strategy of LysM-cre *Cfp*^fl/fl^ mice, and LysM-cre *Cfp*^fl/fl^ had similar rCBF with *Cfp*^fl/fl^ mice.**

(a) The gene targeting strategy for LysM-cre *Cfp*^fl/fl^ mice.

(b) PCR used for genotyping LysM-cre *Cfp*^fl/fl^ and *Cfp*^fl/fl^ mice. LysM-cre *Cfp*^fl/fl^ mice produced both an ~700bp amplicon and an ~241bp amplicon. *Cfp*^fl/fl^ mice produced only the ~241bp amplicon.

(c) Assessment of rCBF in LysM-cre *Cfp*^fl/fl^ and *Cfp*^fl/fl^ mice subjected to tMCAO.
